# Retraction Note to: Multifocal multi-organ ischaemia and infarction in a preterm baby due to maternal intravenous cocaine use: a case report

**DOI:** 10.1186/s13256-016-1022-4

**Published:** 2016-09-05

**Authors:** Ben C. Reynolds, Dawn M. K. Penman, Allan G. Howatson, Lesley A. Jackson, Charles H. Skeoch

**Affiliations:** 1 Neonatal Unit, Princess Royal Maternity Hospital, Alexandra Parade, Glasgow, UK; 2 Department of Paediatric Pathology, Royal Hospital for Sick Children, Dalnair Street, Glasgow, UK

## Retraction note

This article [1] has been retracted by the publisher because it was republished in the journal [2] due to an error during transfer of the journal between publishers in 2009. BioMed Central apologizes to the authors and readers for this error and for any inconvenience caused.
